# 3D Porous Ti_3_C_2_ MXene/NiCo-MOF Composites for Enhanced Lithium Storage

**DOI:** 10.3390/nano10040695

**Published:** 2020-04-07

**Authors:** Yijun Liu, Ying He, Elif Vargun, Tomas Plachy, Petr Saha, Qilin Cheng

**Affiliations:** 1 Key Laboratory for Ultrafine Materials of Ministry of Education, School of Materials Science and Engineering, East China University of Science and Technology, Shanghai 200237, China; lyjecust@126.com; 2 Sino-EU Joint Laboratory of New Energy Materials and Devices, Tomas Bata University in Zlin, 760 01 Zlin, Czech Republic; elifvargun@gmail.com (E.V.); plachy@utb.cz (T.P.); saha@utb.cz (P.S.); 3 Department of Chemistry, Mugla Sitki Kocman University, 48000 Mugla, Turkey

**Keywords:** MXene, NiCo-MOF, 3D porous composite, lithium ion batteries

## Abstract

To improve Li storage capacity and the structural stability of Ti_3_C_2_ MXene-based electrode materials for lithium-ion batteries (LIBs), a facile strategy is developed to construct three-dimensional (3D) hierarchical porous Ti_3_C_2_/bimetal-organic framework (NiCo-MOF) nanoarchitectures as anodes for high-performance LIBs. 2D Ti_3_C_2_ nanosheets are coupled with NiCo-MOF nanoflakes induced by hydrogen bonds to form 3D Ti_3_C_2_/NiCo-MOF composite films through vacuum-assisted filtration technology. The morphology and electrochemical properties of Ti_3_C_2_/NiCo-MOF are influenced by the mass ratio of MOF to Ti_3_C_2_. Owing to the interconnected porous structures with a high specific surface area, rapid charge transfer process, and Li^+^ diffusion rate, the Ti_3_C_2_/NiCo-MOF-0.4 electrode delivers a high reversible capacity of 402 mAh g^−1^ at 0.1 A g^−1^ after 300 cycles; excellent rate performance (256 mAh g^−1^ at 1 A g^−1^); and long-term stability with a capacity retention of 85.7% even after 400 cycles at a high current density, much higher than pristine Ti_3_C_2_ MXene. The results highlight that Ti_3_C_2_/NiCo-MOF have great potential in the development of high-performance energy storage devices.

## 1. Introduction

With the ever-growing energy shortage and environmental pollution, the exploration of advanced renewable energy technologies has become extremely imperative [1]. Among various energy storage devices, lithium-ion batteries (LIBs) have been regarded as very attractive candidates for portable electronic devices and low-emission electric vehicles owing to their high energy density, long-term cyclability, and environmental benignity [2,3,4,5]. Currently, graphite is a commonly-used anode of commercial LIBs, however, it cannot satisfy the burgeoning demand of next-generation energy storage systems with high rate and large capacity owing to its low specific capacity [6]. Hence, much effort should be focused on the development of new electrode materials with rational nanoarchitectures and desired components.

Recently, the application of a fascinating family of 2D metal carbides/nitrides, known as MXene, has been highlighted in the catalysis [7], sensors [8], supercapacitors [9,10], and Li-ion batteries [11,12] because of its metallic conductivity, hydrophilic surface, and adjustable interlayer spacing [13]. In this regard, Ti_3_C_2_ MXene is the most extensively used as electrode materials for energy storage devices for its low Li^+^ diffusion barrier and accessibility [14,15,16]. Nevertheless, analogously to other 2D layered materials, the inevitable self-stacking of Ti_3_C_2_ MXene flakes during electrode fabrication process usually results in limited accessibility to electrolyte ions and low utilization of electrochemical active sites, which significantly deteriorate the capacity and rate performance of MXene-based electrode materials. In order to address this issue, different strategies including creating porous structures [17], introducing interlayer spacers [18], and constructing 3D macroporous frameworks [19] have been developed so far. In spite of considerable progress in the development of Ti_3_C_2_ MXene, its practical application in LIBs is still restricted to a certain extent. This is because the introduction of some spacers [20,21] into MXene sheets reduces the conductivity and specific surface area of the MXene electrode, and the volume change during the electrochemical reaction could not be alleviated effectively on account of a weak interaction between MXene and other active materials. To this end, it is a pressing task to construct a 3D hierarchical porous structure using proper building blocks to fabricate MXene-based composite electrodes with enhanced Li-ion storage capacity and cycling lifespan.

On the other hand, as a novel class of porous materials, metal organic frameworks (MOFs) possess high surface area, ultrahigh porosity, structural tailorability, and highly ordered structures [22,23], which allow them to facilitate fast mass and electron transportation for Li-ion batteries. In particular, recent studies indicate that MOFs with appropriate organic ligand and metal ions can achieve both high stability and electrochemical performance [24]. For instance, Co-MOF synthesized by the reaction of benzenedicarboxylic acid (BDC) with Co^2+^ delivered a large reversible capacity of 650 mAh g^−1^ at a current density of 50 mA g^−1^ after 100 cycles and excellent cycling stability [25]. Moreover, a 2D ultrathin NiCo-MOF catalyst with BDC ligand exhibited high electrocatalytic activity and long-term catalytic stability [26]. In view of the fact that NiCo-MOF nanosheets have high active surface area, unsaturated metal sites, rapid electron transfer, and short diffusion paths of ions, it is beneficial to design MXene/NiCo-MOF composite architectures rationally as anodes for LIBs, in order to take full advantages of both components.

Following this idea, herein, an effective strategy is developed to prepare 3D porous Ti_3_C_2_/NiCo-MOF composites via an interlayer hydrogen-bond interaction between Ti_3_C_2_ and NiCo-MOF nanosheets. Benefitting from the synergistic effect of Ti_3_C_2_ MXene and MOF, the formed 3D interconnected porous architectures not only efficiently restrain self-restacking of both Ti_3_C_2_ and MOF flakes, but also accelerate the transportation of ion/electron. As a result, the Ti_3_C_2_/NiCo-MOF composite electrode delivers a reversible capacity of 402 mAh g^−1^ at 0.1 A g^−1^ after 300 cycles, as well as excellent rate capability and cycling performance. In addition, the effect of the NiCo-MOF loading in the composites on the structural and electrochemical properties of the composite is also investigated.

## 2. Experimental

### 2.1. Materials

Ti_3_AlC_2_ powder (≥98% purity, 200 mesh) was commercially available from Forsman Scientific (Beijing, China) Co., Ltd. Hydrofluoric acid (≥40%), lithium hydroxide, and ethnol (≥99.7%) were all obtained from Shanghai Titan Scientific Co., Ltd. (Shanghai, China). CoCl_2_·6H_2_O (≥99%), NiCl_2_·6H_2_O (≥99%), triethylamine (TEA, ≥98%), *N*,*N*-dimethylformamide (DMF), and *N*-methyl-2-pyrrolidone (NMP) were all obtained from Shanghai Aladdin Bio-Chem Technology Co., Ltd. (Shanghai, China). Benzenedicarboxylic acid (BDC) was purchased from Shanghai Macklin Biochemical Co., Ltd. (Shanghai, China). All chemicals were directly used without any further treatment.

### 2.2. Synthesis of Ti_3_C_2_ MXene Nanosheets

Firstly, 1 g of Ti_3_AlC_2_ powder was immersed in 50 mL of 40% hydrofluoric acid (HF) solution at 50 °C by stirring for 24 h to remove the Al layers [27]. Afterwards, the solid residue was washed with deionized water and then centrifuged at 8000 rpm several times until the pH of the supernatant was above 6, and then the wet sediments were subjected to vacuum freeze-drying steps to obtain the dry Ti_3_C_2_ powder. Subsequently, the Ti_3_C_2_ powder was dispersed in 1 M LiOH solution for 24 h at 40 °C, then the alkalized Ti_3_C_2_ (alk-Ti_3_C_2_) was centrifuged and washed by deionized water several times until the pH of the solution was 8−9. The obtained sample was also dried by vacuum freeze-drying. Finally, in order to delaminate Ti_3_C_2_, 200 mg of the alk-Ti_3_C_2_ was added into 200 mL deionized water and sonicated for 4 h. This mixture was centrifuged at 3500 rpm for 0.5 h, and then the supernatant was collected. The concentration of the Ti_3_C_2_ sheets was determined by filtering a known volume of the delaminated Ti_3_C_2_ suspension through a Millipore membrane (*d* = 0.2 um) and measuring the weight of the film after vacuum drying.

### 2.3. Synthesis of Ultrathin NiCo-MOF Nanosheets

NiCo-MOF nanosheets were synthesized according to the previous report [26] with minor modification. Typically, 2 mL of ethanol, 2 mL of distilled water, and 32 mL of DMF were mixed in a 100 mL beaker. Then, BDC (0.75 mmol) was dissolved into the above solution under ultrasonication. Subsequently, NiCl_2_·6H_2_O (0.375 mmol) and CoCl_2_·6H_2_O (0.375 mmol) were added with continuous stirring. After that, 0.8 mL of TEA was quickly added into the mixed solution under stirring for 20 min. Afterwards, the suspension remained stationary for 8 h at room temperature. Finally, the products were collected after a centrifugation, washing with water, and drying in a vacuum oven.

### 2.4. Fabrication of 3D Porous Ti_3_C_2_/NiCo-MOF Composites

Ti_3_C_2_/NiCo-MOF composites were synthesized via a vacuum-assisted filtering process. First, 40 mg of NiCo-MOF powder was dispersed into the delaminated Ti_3_C_2_ aqueous solution (200 mL, 0.5 mg/mL) under sonication for 4 h, and filtered through a Millipore membrane (0.2 um pore size) to obtain the composite film. Then, the sample was dried by vacuum freeze-drying method. The resultant composite films were denoted as Ti_3_C_2_/NiCo-MOF-x, where x was the mass ratios of NiCo-MOF to Ti_3_C_2_, that is, 0.2, 0.3, 0.4, and 0.5, respectively. In addition, Ti_3_C_2_ nanosheet films were also prepared for comparison.

### 2.5. Material Characterization

The crystallographic structure of the materials was determined through a Bruker D8 Advance X-ray powder diffractometer (Rigaku Corporation, Tokyo, Japan) with Cu Kα radiation (λ = 0.154 nm). The microstructure of the samples was observed using field emission scanning electron microscopy (FESEM, Hitachi S4800, Ibaraki, Japan) and transmission electron microscopy (TEM, JEOL JEM-2100, Ibaraki, Japan), respectively. Energy-dispersive X-ray spectroscopy (EDS) was carried out on a Quantax 400-30 (Beuker AXS Gmbh, Karlsruhe, Germany). N_2_ adsorption- desorption isotherms of the materials were performed with a JW-BK112T (Beijing JWGB Sci.& Tech. Co., Ltd, Beijing, China) analyzed at 77 K. The total specific surface area (SBET) was deduced by the multi-point Brunauer-Emmett-Teller (BET) method. Element composition and surface properties were investigated by X-ray photoelectron spectroscopy (XPS, ESCALAB 250Xi, Basingstoke, UK).

### 2.6. Electrochemical Measurements

Coin-type 2032 cells were fabricated for electrochemical tests. The working electrode was fabricated by mixing an active material (80 wt%), acetylene black (10 wt%), and poly (vinyl difluoride) (PVDF, 10 wt%) in NMP to form a slurry that was then coated onto copper foil and dried in a vacuum oven at 120 °C for 12 h. The electrolyte was 1 M LiPF_6_ in a mixture of ethylene carbonate, diethyl carbonate, and dimethyl carbonate (1:1:1 vol%). The cells were assembled in an argon-filled glovebox with a Celgard 2400 polyethylene membrane as the separator. The mass loading of the active material was about 1.0 mg cm^−2^. The galvanostatic charge/discharge cycling tests were conducted on LAND CT2001A battery testing system. Cyclic voltammetry (CV) and electrochemical impedance spectroscopy (EIS) were carried out using a CHI660E electrochemical station. CV data were collected at a scan rate of 0.1 mV s^−1^ between 0.01 and 3.0 V, while the EIS data were recorded over a frequency range from 0.01 Hz to 100 kHz with a potential amplitude of 5 mV.

## 3. Results and Discussion

**Figure 1:**
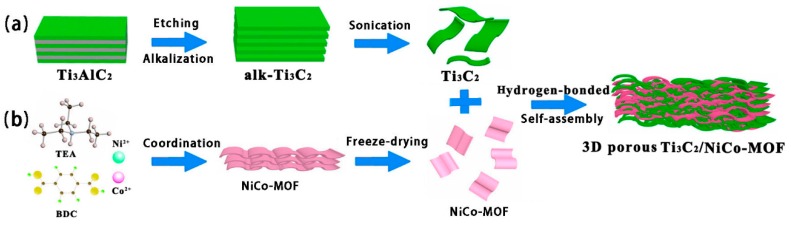
Schematic illustration of the preparation process of Ti_3_C_2_/NiCo-MOF. (**a**) Fabrication process of Ti_3_C_2_ nanosheets, (**b**) Preparation process of NiCo-MOF nanosheets.

Figure 1 schematically illustrates the preparation process of 3D porous Ti_3_C_2_/NiCo-MOF composites via self-assembly induced by hydrogen bonding. First, 2D NiCo-MOF nanosheets are synthesized at room temperature through coordination interaction between bimetallic ions (Ni^2+^, Co^2+^) and BDC ligands, in which both Ni and Co atoms are coordinated octahedrally by six O atoms for the generation of 2D bimetal layers separated by BDC molecules [26], leading to plenty of −COOH groups on the MOF nanosheets. Then, multilayered Ti_3_C_2_ MXene with enlarged interlayer spacing is obtained by etching Ti_3_AlC_2_ with HF solution followed by alkalization, and subsequent exfoliation through sonication results in the formation of Ti_3_C_2_ nanosheets whose surface is anchored by large amount of terminal groups (−F, −O, and −OH). Thus, when NiCo-MOF was added into Ti_3_C_2_ nanosheets solution, the Ti_3_C_2_/NiCo-MOF composite film with interconnected porous structure was naturally constructed owing to the interlayer hydrogen bonds between MXene nanosheets and MOF nanosheets upon vacuum-assisted filtration.

**Figure 2:**
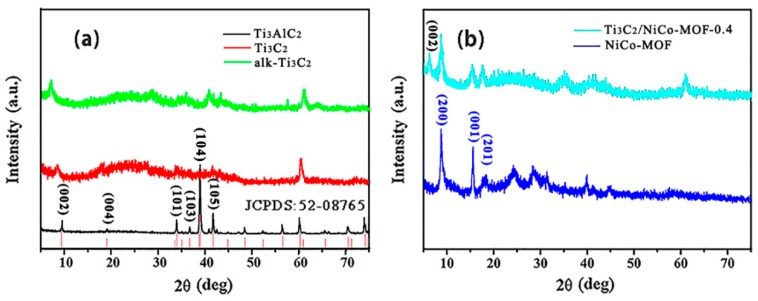
(**a**) XRD patterns of Ti_3_AlC_2_, Ti_3_C_2_, and alk-Ti_3_C_2_; (**b**) XRD patterns of NiCo-MOF and Ti_3_C_2_/NiCo-MOF-0.4.

X-ray diffraction (XRD) patterns of Ti_3_AlC_2_, Ti_3_C_2_, alk-Ti_3_C_2_, NiCo-MOF, and Ti_3_C_2_/NiCo- MOF-0.4 are illustrated in Figure 2. Clearly, typical diffraction peaks corresponding to Ti_3_AlC_2_ phase ((JCPDS) card No. 52-0875) can be observed in Figure 2a. After HF etching of Ti_3_AlC_2_, the sharp (104) diffraction peak at around 39° almost vanishes, suggesting a successful transformation from Ti_3_AlC_2_ to Ti_3_C_2_ caused by the removal of etched Al layers [28]. Simultaneously, a strong (002) peak shifts from 9.52° for Ti_3_AlC_2_ to 8.87° for Ti_3_C_2_ MXene with an increasing of the interlayer spacing from 0.93 to 1.0 nm. Recent studies have indicated that the interlayer spacing can be further enlarged after treatment of Ti_3_C_2_ with strong alkaline solution [29]. Therefore, when using LiOH as an alkalizer in our experiment, the distance between the layers of Ti_3_C_2_ is efficiently expanded, as evidenced by the XRD pattern of alk-Ti_3_C_2_ in which the (002) peak shifts to 7.27° (*d* = 1.22 nm). The alkalization process facilitates the delamination of Ti_3_C_2_ and the intercalation of Li^+^. Figure 2b depicts the crystal features of NiCo-MOF and Ti_3_C_2_/NiCo-MOF. NiCo-MOF exhibits three main peaks at 8.8°, 15.5°, and 18.2° that are indexed to (200), (001), and (201) planes, respectively, which are typical characteristics of NiCo-MOF ultrathin nanosheets synthesized with BDC ligands [26,30]. After combination with Ti_3_C_2_ nanosheets, the diffraction peaks generated by the Ti_3_C_2_/NiCo-MOF composite are similar to those from pure NiCo-MOF and Ti_3_C_2_ MXene, indicating that the presence of the MXene nanosheets has little effect on the crystal structure of the NiCo-MOF. However, the (002) peak for Ti_3_C_2_/NiCo-MOF composite shifts to lower angle 6.3° compared with that of alk-Ti_3_C_2_ in Figure 2a. The further increased interlayer spacing strongly suggests that MOF nanosheets interleave Ti_3_C_2_ layers to effectively overcome the self-restacking of MOF or Ti_3_C_2_ flakes, which in turn provides easy access for electrolyte ions during electrochemical reaction and guarantees high-rate capability.

**Figure 3:**
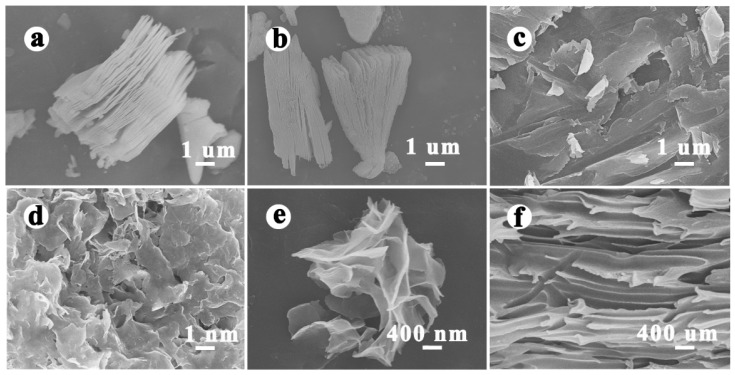
Scanning electron microscopy (SEM) images of (**a**) Ti_3_C_2_, (**b**) alk-Ti_3_C_2_, (**c**) exfoliated Ti_3_C_2_, (**d**) NiCo-MOF, and (**e**) Ti_3_C_2_/NiCo-MOF-0.4. (**f**) Cross-sectional SEM image of Ti_3_C_2_/NiCo-MOF-0.4.

The morphology and microstructure of Ti_3_C_2_, alk-Ti_3_C_2_, exfoliated Ti_3_C_2_, NiCo-MOF, and Ti_3_C_2_/NiCo-MOF-0.4 were characterized by SEM. As shown in Figure 3a, the etched Ti_3_C_2_ MXene exhibits an accordion-like multilayered architecture composed of individual nanoflakes, with the spacing between flakes ranging from tens of nanometers to hundreds of nanometers. After the treatment with LiOH solution, the obtained alk-Ti_3_C_2_ still preserves a multilayered structure (Figure 2b), but the alkalization process enlarges the interlayer spacing remarkably and is very favorable for the subsequent delamination. As the suspension of alk-Ti_3_C_2_ subjected to ultrasonication, 2D lamellar structures of Ti_3_C_2_ nearly disappear and ultrathin nanosheets with a lateral size of several micrometers can be clearly observed in Figure 3c, demonstrating a successful exfoliation of layered Ti_3_C_2_. A similar ultrathin morphology is also detected for the as-synthesized NiCo-MOF, which has a smaller lateral dimension than Ti_3_C_2_ sheets (Figure 3d). However, after coupling with Ti_3_C_2_ nanoflakes induced by the hydrogen-bond interaction, the Ti_3_C_2_/NiCo-MOF composite exhibits 3D hierarchical architectures assembled by NiCo-MOF and Ti_3_C_2_ nanosheets (Figure 3e). The interlaced nanosheets are tightly attached to form interconnected porous networks for fast charge storage and also prevent the self-restacking of both sheets. Moreover, the cross-sectional SEM image (Figure 3f) confirms the layered structure of the composite film with alternating Ti_3_C_2_ and MOF nanosheets layers.

**Figure 4:**
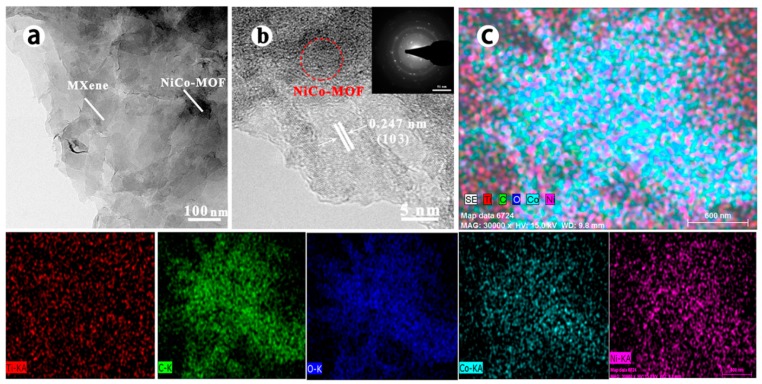
(**a**) Transmission electron microscopy (TEM) image of Ti_3_C_2_/NiCo-MOF-0.4; (**b**) high-resolution TEM image of Ti_3_C_2_/NiCo-MOF-0.4, the inset is the selected area electron diffraction (SAED) pattern of Ti_3_C_2_; and (**c**) mapping image of Ti_3_C_2_/NiCo-MOF-0.4.

The detailed microstructure of the Ti_3_C_2_/NiCo-MOF-0.4 composite is further investigated by TEM. As shown in Figure 4, Ti_3_C_2_ MXene has a larger lateral size than NiCo-MOF, which is in agreement with the SEM observation in Figure 3c,d. Small-sized NiCo-MOF sheets adhere to the surface of large MXene flakes to form a hierarchical structure, as verified by a sharp contrast between the both components (Figure 4a). Meanwhile, the lattice fringes are very visible in high resolution transmission electron microscopy (HRTEM) image and lattice spacing assigned to (103) plane is about 0.247 nm (Figure 4b), which is in accordance with that of MXene phase [28]. Meanwhile, NiCo-MOF does not show distinct crystal lattice as expectation owing to its low crystallinity. The selected area electron diffraction (SAED) pattern (inset in Figure 4b) reveals the high crystallinity of Ti_3_C_2_. The EDS elemental mapping (Figure 4c) indicates the homogeneous distribution of Ti, C, O, Co, and Ni elements in the Ti_3_C_2_/NiCo-MOF nanocomposites, which demonstrates that NiCo-MOF sheets uniformly integrate with Ti_3_C_2_ sheets. In addition, to confirm the structural advantages of the porous Ti_3_C_2_/NiCo-MOF composite, N_2_ isotherm was employed to measure the specific surface area. The Ti_3_C_2_/NiCo-MOF-0.4 composite possesses a higher BET surface area of 60.3 m^2^ g^−1^ than that of MXene (23.5 m^2^ g^−1^) or NiCo-MOF (37.1 m^2^ g^−1^) [31]. The increased surface area caused by 3D porous structures and expanded interlayer spacing between Ti_3_C_2_ sheets could offer rapid infiltration of electrolyte and more active sites for electrochemical reaction.

**Figure 5:**
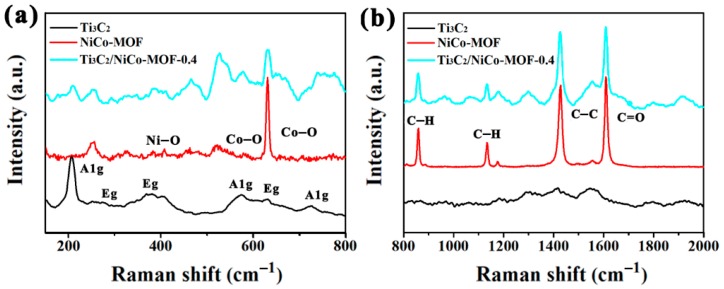
Raman spectra of Ti_3_C_2_, NiCo-MOF, and Ti_3_C_2_/NiCo-MOF-0.4. (**a**) low Raman shift range, (**b**) high Raman shift range.

Raman spectra were also performed to verify the surface structure of Ti_3_C_2_, NiCo-MOF, and Ti_3_C_2_/NiCo-MOF-0.4. As can be found from Figure 5a, pure Ti_3_C_2_ MXene exhibits typical Raman peaks. In particular, the peaks at 203, 575, and 719 cm^−1^ are attributed to A1g symmetry out-of-plane vibrations of Ti and C atoms, respectively, while those at 282, 365, and 622 cm^−1^ correspond to the Eg group vibrations, including in-plane (shear) modes of Ti, C, and surface functional group atoms [32]. While for pristine NiCo-MOF, the peak at 415 cm^−1^ is assigned to Ni−O, and the two peaks at 526 and 630 cm^−1^ correspond to Co−O stretching vibration [33,34], as well as others at 1423 and 1607 cm^−1^ corresponding to C−C and C=O vibration (Figure 5b), respectively. In addition, the peaks at 860, 1136, and 1175 cm^−1^ can be ascribed to the deformation modes of the C−H groups, which are also the characteristics peaks of the NiCo-MOF [35]. Note that the aforementioned peaks of Ti_3_C_2_ and NiCo-MOF appear in the Ti_3_C_2_/NiCo-MOF-0.4, indicating the co-existence of NiCo-MOF and Ti_3_C_2_ in the composite.

**Figure 6:**
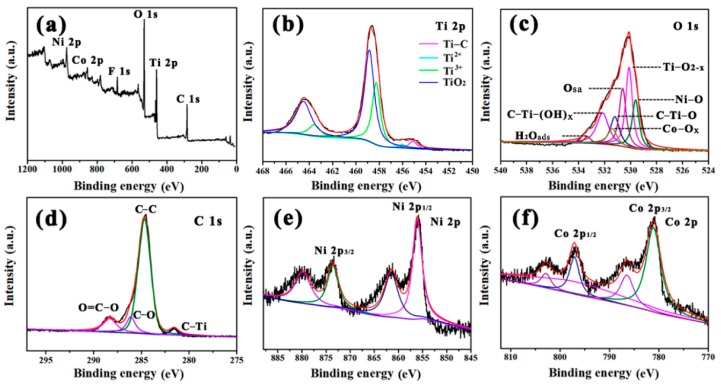
(**a**) X-ray photoelectron spectroscopy (XPS) survey spectrum of the Ti_3_C_2_/NiCo-MOF-0.4, (**b**) Ti 2p spectra, (**c**) O 1s spectra, (**d**) Ni 2p spectra, (**e**) C 1s spectra, and (**f**) Co 2p spectra.

XPS measurement was carried out to investigate the surface electronic states of Ti_3_C_2_/NiCo-MOF-0.4. The XPS survey spectrum presented in Figure 6a suggests the presence of C, Ti, O, F, Co, and Ni elements in the composite, in which F, Ti, and C elements come from Ti_3_C_2_ after HF etching, while Co, Ni, and O elements originate from NiCo-MOF. High-resolution XPS spectra of Ti 2p (Figure 6b) can be deconvoluted into four pairs of doublets for Ti−C (455.4/461 eV), Ti^2+^ (456/461.3 eV), Ti^3+^ (458.1/463.4 eV), and TiO_2_ (458.9/464.5 eV) [36]. As shown in Figure 6c, seven peaks of O 1s XPS spectra attributed to the surface Ni−O, Ti−O_2−*x*_, O_sa_ (surface active oxygen), C−Ti−O_x_, Co−O, C−Ti−(OH)*_x_* (or O=C−O), and H_2_O_ads_ (adsorbed water) species are centered at 529.6, 530.1, 530.6, 531.2, 531.4, 532.1, and 533.3 eV, respectively [37,38]. The Ti 2p and O 1s results indicate that the Ti_3_C_2_ is partially oxidized to TiO_2_ owing to more defective and large exposed surface of nanosheets. However, considering the fact that no obvious peaks related to TiO_2_ can be detected in the XRD pattern of Ti_3_C_2_/NiCo-MOF-0.4, this partial oxidation reaction possibly only takes place on the contact surface of Ti_3_C_2_ and NiCo-MOF in view of an effective surface analysis method of XPS measurement. As for the C 1s core level spectra (Figure 6d), it can be fitted with four peaks located at 281.6 (C−Ti), 284.6 (C−C), 286.1 (C−O), and 288.3 eV (O=C−O), respectively. For Ni 2p and Co 2p spectra in Figure 6e, f, two peaks of Co 2p_1/2_ (796.8 eV) and Co 2p_3/2_ (780.2 eV) along with satellite peaks at 785.1 and 802.6 eV are observed, while the major peaks at 856.1 and 873.6 eV are assigned to Ni 2p_1/2_ and Ni 2p_3/2_, respectively, which coincide with the reported value [39,40].

**Figure 7:**
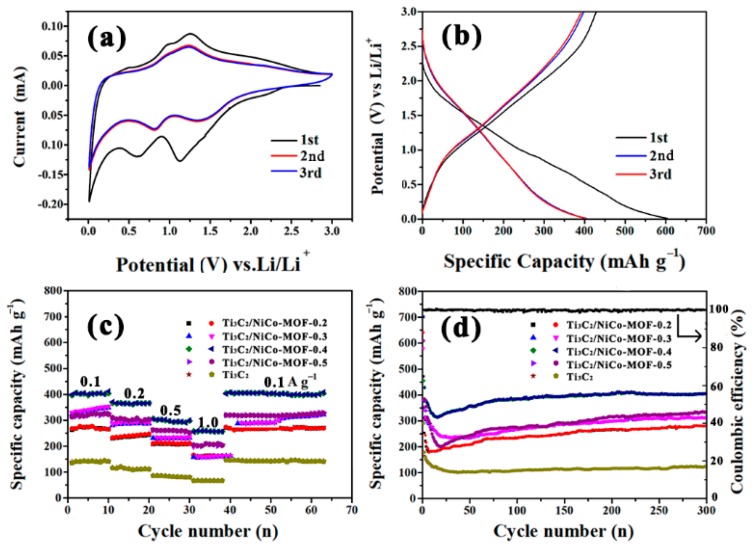
(**a**) Cyclic voltammetry (CV) curves of Ti_3_C_2_/NiCo-MOF-0.4 composites at a scanning rate of 0.1 mV s^−1^, (**b**) charge-discharge curves of the Ti_3_C_2_/NiCo-MOF-0.4 composites at a current density of 0.1 A g^−1^ for the initial three cycles, (**c**) rate performance of Ti_3_C_2_/NiCo-MOF composites and Ti_3_C_2_ at various current densities, and (**d**) cycling performance of Ti_3_C_2_/NiCo-MOF composites and Ti_3_C_2_ at a current density of 0.1 A g^−1^.

In order to further evaluate the lithium storage performance of the prepared composites, the CV profiles of Ti_3_C_2_/NiCo-MOF-0.4 as anode material for LIBs are given in Figure 7a. It can be found that, in the first turn of the CV curve, two peaks are easily observed—the first peak at around 1.12 V corresponds to the irreversible solid electrolyte interphase (SEI) formation [41], while another peak around 0.6 V can be attributed to the trapping of Li^+^ between Ti_3_C_2_ and NiCo-MOF nanosheets [19]. During the subsequent cycles, a broad oxidation reversible peaks located at 1.24 V may be caused by the extraction of Li^+^ from Ti_3_C_2_ and NiCo-MOF nanosheets. In the second discharge cycle, the two cathodic peaks at 0.81 and 1.41 V are related to the reduction of Co^2+^ and Ni^2+^ to metallic Co and Ni, respectively [38], as well as partial insertion of Li^+^ in Ti_3_C_2_, and the peak shift is the result of the irreversible reaction during the first charge-discharge cycle. The CV curves of the second and third circles are highly coincident, indicating that the Ti_3_C_2_/NiCo-MOF-0.4 electrode is highly reversible in the electrochemical reaction process. Figure 7b shows the charge/discharge curves for the first three cycles of the Ti_3_C_2_/NiCo-MOF-0.4 at 0.1 A g^−1^. During the first cycle, Ti_3_C_2_/NiCo-MOF-0.4 delivers a high discharge and charge capacity of 603.6 and 428.8 mAh g^−1^, respectively. The capacity loss is the result of the formation of SEI film, which is in agreement with CV results. Nevertheless, the almost overlapped discharge and charge curves of Ti_3_C_2_/NiCo-MOF-0.4 in the second and third cycles demonstrate the good reversibility and stability of the composite electrode.

The rate performance of Ti_3_C_2_/NiCo-MOF composites and Ti_3_C_2_ electrodes is presented in Figure 7c. It is obvious that the Ti_3_C_2_ electrode exhibits a charge capacity of 141, 116, 85, and 67 mAh g^−1^ at a current density of 0.1, 0.2, 0.5, and 1 A g^−1^, respectively. The relatively low capacity results from the slow Li ions diffusion limited by compact stacking of multi-layer Ti_3_C_2_. In the case of Ti_3_C_2_/NiCo-MOF composites, their electrochemical performance depends largely on the NiCo-MOF loading in the composite. The specific capacity of the Ti_3_C_2_/NiCo-MOF composites increases to the maximum and then decreases with the increase of NiCo-MOF content, that is, Ti_3_C_2_/NiCo-MOF-0.4 exhibits the highest capacity at the same current density compared with Ti_3_C_2_ and other composite electrodes. Specially, for the Ti_3_C_2_/NiCo-MOF-0.4 electrode, a discharge capacity of 402, 366, 303, and 256 mAh g^−1^ can be obtained at 0.1, 0.2, 0.5, and 1 A g^−1^, respectively. As the current density goes back to 0.1 A g^−1^, the capacity almost recovers its initial value, indicating excellent rate performance of the Ti_3_C_2_/NiCo-MOF-0.4 electrode.

The cycling performances of Ti_3_C_2_/NiCo-MOF composites and Ti_3_C_2_ at a current density of 0.1 A g^−1^ are summarized in Figure 7d. Apparently, the capacity of Ti_3_C_2_ shows a downward trend in the initial 30 cycles and remains at around 110 mAh g^−1^ in 300 cycles. In contrast, that of the Ti_3_C_2_/NiCo-MOF composites decreases rapidly before 20 cycles, and then increases gradually and remains stable in the following cycling process. An initial decline in the capacity could be associated with the irreversible reaction between Ti_3_C_2_/NiCo-MOF nanosheets and the electrolyte and lithiation-induced mechanical degradation, while a subsequent increase in the capacity reveals a significant lithium-induced reactivation of the composite electrodes [42]. As expected, Ti_3_C_2_/NiCo-MOF-0.4 exhibits superior cycling stability. It delivers a discharge capacity of 609 mAh g^−1^ and charge capacity of 440 mAh g^−1^ in the first cycle, respectively. The corresponding coulombic efficiency is 72.2% and nearly reaches 100% afterwards. After 300 cycles, the Ti_3_C_2_/NiCo-MOF-0.4 electrode achieves a capacity of 402 mAh g^−1^, much higher than all electrodes. The above results clearly indicate enhanced Li storage of Ti_3_C_2_/NiCo-MOF-0.4 in the aspect of both high capacity and excellent cycling performance, which may be connected with the 3D porous interpenetrating frameworks and enhanced electrical conductivity aroused by the coupling effect of NiCo-MOF and Ti_3_C_2_. As far as we know, the surface of Ti_3_C_2_ nanosheets anchored by −F, −O, and −OH groups after HF etching and alkalization actually impedes Li^+^ transport and reduces the conductivity of Ti_3_C_2_. After integrating with NiCo-MOF flakes, these groups from Ti_3_C_2_ could be bonded to hydrogen atom in −COOH from MOF to construct 3D porous Ti_3_C_2_/NiCo-MOF composites, facilitating accessibility of composite nanosheets to the electrolyte ions. Moreover, with increasing mass ratio of NiCo-MOF to Ti_3_C_2_ MXene (e.g., from 0.1 to 0.4), the specific surface area, and the interlayer spacing of composites also increase, which undoubtedly improve active sites for electrochemical reaction and speed up diffusion and transport of ions, thus leading to superior electrochemical performance of Ti_3_C_2_/NiCo-MOF-0.4 composite. As the mass ratio is further increased up to 0.5, the excessive MOF sheets with poor conductivity increase the internal resistance of composite electrode and result in performance degradation of Ti_3_C_2_/NiCo-MOF-0.5. Therefore, appropriate NiCo-MOF content in the composites is essential to achieve the optimal electrochemical performance.

**Figure 8:**
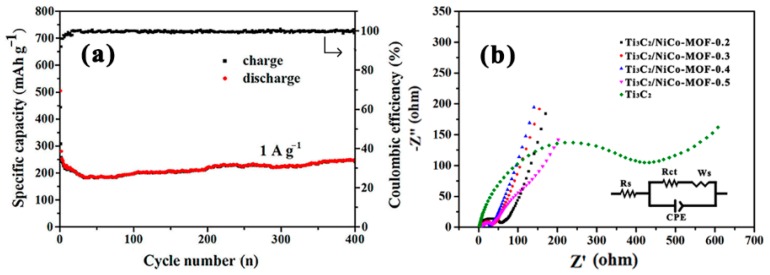
(**a**) Long cycling stability curves of Ti_3_C_2_/NiCo-MOF-0.4 at 1 A g^−1^, (**b**) the Nyquist plots of Ti_3_C_2_ and Ti_3_C_2_/NiCo-MOF composite electrodes. The inset is an equivalent circuit model.

To highlight the role of NiCo-MOF in acquiring enhanced Li storage performance of Ti_3_C_2_/NiCo-MOF composite electrodes, the long-term stability of the Ti_3_C_2_/NiCo-MOF-0.4 electrode at a high current density of 1 A g^−1^ is explored. As illustrated in Figure 8a, a high discharge capacity of 504.5 mAh g^−1^ can be reached at the first cycle. Then, the Ti_3_C_2_/NiCo-MOF-0.4 electrode exhibits a slightly increased capacity after the initial 30 cycles and remains at a relatively high capacity of 240 mAh g^−1^ along with a capacity retention of 85.7% even after 400 cycles, verifying its excellent long cycling life at a high rate as well. Incorporation of NiCo-MOF nanosheets into the interlayers of Ti_3_C_2_ MXene results in the formation of a porous interconnected architecture, which endows the Ti_3_C_2_/NiCo-MOF-0.4 electrode with robust structural integrity to withstand the volume changes during the fast charge-discharge process, thus ensuring its high-rate capability and long cycling durability.

The prominent performance of Ti_3_C_2_/NiCo-MOF-0.4 electrode materials for LIBs can be confirmed by EIS measurement. Figure 8b displays the Nyquist plots of the Ti_3_C_2_ and Ti_3_C_2_/NiCo-MOF electrodes. The inset in Figure 8b is an equivalent circuit model that includes bulk electrolyte resistance (*R*_s_), the charge transfer resistance *R*_ct_, and the Warburg resistance (*W*_s_) related to Li^+^ ions diffusion in the bulk electrode [43]. As shown in the Nyquist plots, the depressed semicircle in the medium-to-high frequency region represents *R*_ct_, and an inclined line in the low frequency range corresponds to *W*_s_. The two points that the semicircle intersects the real axis are *R*_s_ and *R*_s_ + *R*_ct_. All the electrodes exhibit similar *R*_s_, while the *R*_ct_ of Ti_3_C_2_/NiCo-MOF-x (x = 0.2, 0.3, 0.4, and 0.5) and Ti_3_C_2_ electrodes is calculated to be 58.3, 39.2, 28.1, 41.9, and 446.2 Ω, respectively. It is evident that Ti_3_C_2_/NiCo-MOF-0.4 shows the lowest charge transfer resistance, demonstrating that the synergistic effect of the NiCo-MOF and Ti_3_C_2_ significantly improves the charge transfer ability of the composite electrode. Additionally, a larger slope of Ti_3_C_2_/NiCo-MOF-0.4 in the low frequency region suggests the greatly reduced Li^+^ diffusion impedance. Both rapid electron and ion transport at the interface and fast Li^+^ diffusion rate into electrode lead to better electrochemical performance of the Ti_3_C_2_/NiCo-MOF-0.4 electrode.

**Figure 9:**
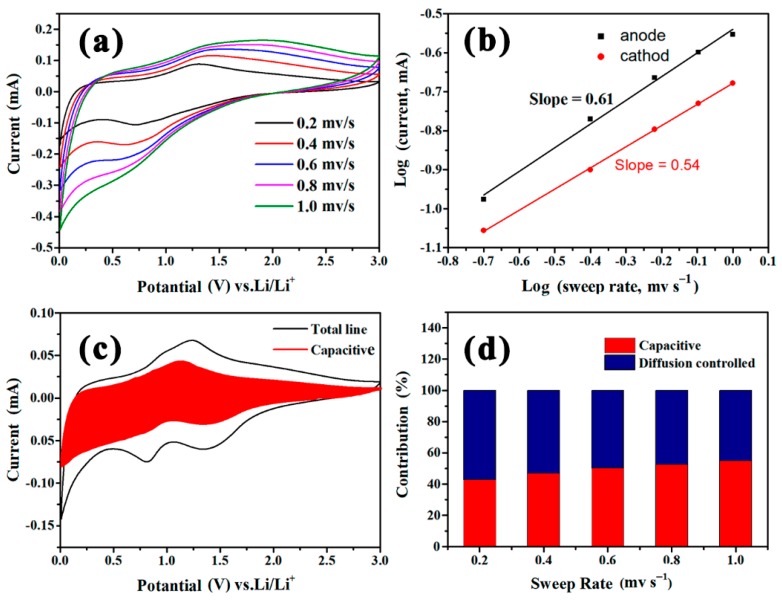
(**a**) CV profiles of the Ti_3_C_2_/NiCo-MOF-0.4 electrode at different scan rates. (**b**) The relationship between peak current and scan rates from 0.2 to 1 mV s^−1^ for the Ti_3_C_2_/NiCo-MOF-0.4 electrode. (**c**) The CV curves of Ti_3_C_2_/NiCo-MOF-0.4 at 0.2 mV s^−1^ with calculated capacitive contribution in the shading. (**d**) The contribution ratio of capacitive and diffusion-controlled capacities of Ti_3_C_2_/NiCo-MOF-0.4 at different scan rates.

To get a better understanding of the electrochemical reaction process, the storage mechanism of the Ti_3_C_2_/NiCo-MOF-0.4 electrode is also analyzed. The CV curves of the Ti_3_C_2_/NiCo-MOF-0.4 electrode recorded at various scan rates from 0.2 to 1.0 mV s^−1^ are plotted in Figure 9a. In general, the variation of current (*i*) with scan rate (*ν*) is represented by the power law of *i* = *aν^b^*, where *a* and *b* are adjustable parameters [44]. The *b* value of 0.5 or 1.0 corresponds to diffusion-controlled process or capacitive behavior, respectively, and it can be calculated by the slope of fitted line of log *i* versus log *ν*. Figure 9b depicts the relationship between log *i* and log *ν* from 0.2 to 1.0 mV s^−1^. The Ti_3_C_2_/NiCo-MOF-0.4 composite possesses *a*, *b* values of 0.61 and 0.54 for the anodic and cathodic peaks, respectively, suggesting that the charge storage process is dominated by diffusion-controlled process, which leads to high capacity of Ti_3_C_2_/NiCo-MOF-0.4 via electron involved redox reaction, as aforementioned [37]. In order to quantitatively determine the ratio of diffusion-controlled and capacitive contribution, a formula of *i* = *k*_1_*ν* + *k*_2_*ν*^1/2^ is applied, where *k*_1_*ν* and *k*_2_*ν* stand for capacitive and diffusion-controlled contributions [45]. On the basis of the analysis of CV curves using this equation, about 42.8% of capacitive contribution can be achieved at the scan rate of 0.2 mV s^−1^ (Figure 9c). Furthermore, it can be found that the capacitive contribution increases from 42.8% to 54.8% with the scan rate from 0.2 to 1.0 mV s^−1^ in Figure 9d, indicating that the Ti_3_C_2_/NiCo-MOF-0.4 composite displays enhanced rate performance.

## 4. Conclusions

In summary, we have adopted a facile ultrasonic and vacuum-assisted filtration method to successfully fabricate 3D Ti_3_C_2_/NiCo-MOF porous composites as anode materials for lithium-ion batteries. The enhanced accessible surface area for electrochemical reaction and expanded interlayer spacing for fast infiltration of Li^+^ into electrodes result in superior electrochemical performance of Ti_3_C_2_/NiCo-MOF composites. The as-prepared Ti_3_C_2_/NiCo-MOF-0.4 electrode exhibits a high reversible capacity of 402 mAh g^−1^ at 0.1 A g^−1^ after 300 cycles, excellent rate performance (256 mAh g^−1^ at 1 A g^−1^), and long-term stability with a capacity retention of 85.7% even after 400 cycles at a high current density. The storage mechanism reveals that the charge storage process is dominated by diffusion-controlled process, which leads to high capacity of the Ti_3_C_2_/NiCo-MOF-0.4 electrode. The present strategy for MXene-based composite nanosheets induced by hydrogen bonds can be extended to other advanced electrodes in energy storage devices with enhanced performance.
